# Digitalis, Etc.

**Published:** 1902-04

**Authors:** C. A. F. Lindorme

**Affiliations:** Atlanta, Ga.


ATLANTA

### Journal-Record of Medicine.

**Successor to Atlanta Medical and Surgical Journal, Established 1855, and Southern Medical Record, Established 1870.**

Vol. IV. APRIL, 1902. No. 1.

BERNARD WOLFF, M.D., EDITOR, Nos. 319-20 Prudential.

Published Monthly.

M. B. HUTCHINS, M.D., BUSINESS MANAGER, No. 64 Marietta St.

### ORIGINAL COMMUNICATIONS.

### DIGITALIS, ETC.

By C. A. F. LINDORME, Ph.D., M.D.,

Atlanta, Ga.

There is nothing more welcome to a theoretician than involuntary endorsement of his theory by practitioners. It is like the hearing of the reciprocal hammering of the two gangs of engineers who started at the opposite points of a mountain, piercing it for a tunnel. The author was delighted at experiencing such satisfaction when he read a number of articles on heart disease, which the *Journal of the American Association* brought in its January 12 number. What Dr. Solomon Solis-Cohen, the first of the clever writers, has most at heart is, to impress the reader with the necessity to pay more attention than is common to other than merely valvular diseases of the heart. But in dwelling upon the frequency of purely muscular lesions, he emphasizes: "Concerning the great importance of the vascular system in diagnosis, prognosis and treatment," "the acute idiopathic dilatation—occurring suddenly or in a comparatively short evolution, from direct strain on the heart." Mentioning among the principal causes of diseases of the cardiac muscle, alcohol and tobacco, he states more particularly the secondary changes of the coats of the blood-vessels as "impairment of cardiac nutrition by ischemia" and "increase of the work placed on the heart to carry on the circulation." Now, if we must accept these theses as practically correct, it follows that the centre of the blood-circulation is not in the heart-muscle, but *in the capillary system.* If William Harvey anticipated three centuries of glory by the satisfaction, "Perpetual movement of the blood in a circle is caused by the beats of the heart," (Curtis, in American Handbook of Physiology) it does not follow that it was the last word to be said in this movement. A mistake it was that in the motion of the blood caused by the beats is to be seen the greatest service of the circulation. The conservation in fresh condition of the blood, it was proved, was not owing to the perpetual movement; it was owing to the physiological condition of the coats of the blood-vessels. The discovery that the cardinal service of the pumping in the heart is in the pumping of oxygen into the capallaries, is rather new. The idea, we think, was put forward the first time in this JOURNAL, in the article, "Heart-Failure," by your humble and obedient servant, but finds endorsement in the above-mentioned articles of the *Journal of the American Association.* If Louis Faugéres Bishop, A.M., M.D., says: "The chief symptom of myocarditis is a failure in the power of the heart to respond to demands of extra work," he must see the leading feature of the thoracic function in the pumping of oxygen merely. As heading the physiology of the region, we have *the chemism of the capillary system.* Hence to a certain degree the heart in its own pathology is a mere accessory. "A great deal depends on the blood-vessels." (Bishop, l. c.) In every pending complication the cardinal question is, how the heart fits the special requirements. "The temporary anemia of a damaged heart through the spasmodic contraction of the coronary arteries may cause a fatal attack of angina, while, had the patient escaped such an accident, he might have lived for years in comparative comfort." (Bishop, l. c.)

J. J. Morrissey, A.M., M.D., in his plea for a more rational prognosis in cardiac affections, says: "A complete diagnosis should be based not so much upon the physical signs, which are frequently deceptive and limited in character, but on a consideration of organs far removed from the thoracic cavity, whose relations, while not intimate, yet are closely affiliated with the results arising from an interference with the circulatory system in consequence of the cardiac disease. Our thoughts should never be centralized on the heart itself to the exclusion of other considerations, which, even more than any conclusions derived from physical signs, may be of paramount importance in aiding us to arrive at a definite prognosis."

Now, the principle which underlies this special declaration reaches a good deal further than the statement of William Harvey. The satisfaction which the latter derived, anatomically, was much too great for physiological extension, even if the age had been prepared for this, which it was not. There are many investigators heavily under the historic influence yet. Our physiological text-books carry a dead weight of hydraulic doctrine, as if, to all intent and purpose, the service of the heart was exhausted with the pumping as such. It is reaching the quick, if J. J. Morrissey says (l. c.): "Intrinsic strength and vitality are not simple qualities, but are complex in character—so complex, indeed, that their consideration means the investigation not alone of the patient himself, but of his progenitors, the diseases of which they died, their power to overcome certain maladies and quickly to succumb to others, the whole question of heredity a topic of such immense ramifications as to be practically forbidden, even in part, in the paper of the limited scope which I have the honor of presenting." The cardinal point, however, from which this modern outlook is taken, is *the cell-life*, and this leads us in the blood-circulation to the capillaries. All that is chemical in the blood-circulation displays its life in the enormous widening of the vesicular lumen, and all that is vital in the heart's action is the accommodation of the hæmoglobin with oxygen. But this is exclusively for the capillary region. Directly the arterial coats need none of the oxygen, nor are the veins incommodated by any surcharge of carbonic acid. The beats of the heart-muscle do not reach, therefore, any further than the capillaries, the rhythmical propulsion of the blood changing into the constant pressure which is furnished by the elasticity of the arterial coats, the extension of the same being in proportion to the resistance offered by the capillary shoals. From this results, however, for therapeutics the important maxim, that it is not by the heart that we reach the capillaries, but by the capillaries that we reach the heart. Nay, by the capillaries we reach a good many other things. I had once a neurasthenic who had gone through the whole rigmarole of soporifics, and had been for two years already suffering the tortures of sleeplessness. I applied massage; I applied it in the evening, and I had the satisfaction to be told the next day by the lady, that she never heard me leave the house, and slept a good round of hours. "Nutrition (or assimilation) reproduction, conduction, and contractility form the important features which we may recognize in all living things, and which we make use of in distinguishing between live and dead matter." (W. H. Howell, in American Textbook of Physiology.) And "recent observers are united on the necessity of oxygen in the working heart." (Curtis, l. c.) Of all this William Harvey did not know anything, and the hydraulic hypothesists do not want to know anything. But the therapeutists are getting to the point ahead of physiology. Bishop says of a case of heart disease (l. c.): "There is no history of specific disease or rheumatism and no abuse of alcohol. The man has worked hard, but apparently not in a way as to impair his health. It is probable that there have been slight arterial changes, which have affected the coronary artery out of proportion with the general arterial system." Now, what is here against the parenthesis which Bishop would have supplied if he had had occasion *suo loco?* That it is not the heart which makes the blood circulation functionate, but the capillaries which make the heart go. It is the osmosis of the capillaries which complete the function of nutrition and synchronously debarrass the body of the effete matter which results from former nutrition. The main of the pertinent economy of the body is perfected accordingly before the whole rhythmical accessory of the heart is called into life, and the service of the rythmical beating of the apex does not extend any further than this accessory genesis; it is the pumping of oxygen, and this exclusively, which the heart is called upon to do; this is the sole purpose of the cardiac mechanism, and it is easy to show experimentally the utter uselessness of the mechanism, if the necessary oxygen is supplied in some other way. Then all pulsation, as well as the lung movement, will stop at once. The thing is, that, in consequence of the closing of the animal organism, the cells of the inner organs are hidden away from the access of air. It must obtain some arrangement, consequently, to procure access to the air as a substitute of the immediate connection with it of the cells in isolated condition. The beating of the heart and the pulmonary circulation are this arrangement.

In this case again, like in so many others in the history of science, the true pioneer is not the physiologist, with his costly laboratory and ulterior institutions, but the general practitioner, the bold therapeutist, the vigilant inquisitive clinician. He sees and watches what it all runs out in; he is by his calling urged to accomplish a task, which the experimenting scientist is liable to neglect over the routine exclusivity of his academic position. Physiology has achieved great things, nobody can deny. But from the admirable analysis of which she may be proud there is a drawback; the detail work with her has been so prominent, so overwhelmingly the ambitious point, that of synthesis of research, not even the first traces can be detected. Our physiological text-books give us wonderful insight into all the single items of the human life. But of what they fail entirely to give us an insight, that is *the life of man.* As a totality, as a unitary organism, the biggest books on physiology do not treat man. Over the vast extent of their analysis they forget entirely the synthesis of the enormous material piled up, and the busy practitioner, if he wants to realize the great advantage which the therapeutist derives from steady physiological genesis of the pathology of the cases before him, must perform the more difficult part of the work himself which, properly detailed, should fall to the lot of the physiologist. Physiology has directly nothing to do with pathology. But if its researches are not made with an eye to medical practice, they will finally become an abstraction monologue which is of no use to anybody. Says Bishop (l. c.): "We have seen in the hospital patients live for months and years with progressive degeneration of the heart, until finally, when they died, it was a sort of wonder how a heart so degenerated could have carried on its work." The explanation is, that if it was not the blood that moved the heart no heart would be able to move the blood. All that the beats of the heart perform is, to keep up the continuity of the oxygen-supply, which, as soon as reaching its destination, the capillaries, takes care of itself, and no less the exchange, the carbonic acid of the body. But if this result is so imperative as to explode undeniably therapeutically, it would for all that be extremely beneficent to see it recognized as general physiological principle. Not all of our therapeutists are pioneers in general practice, and they do well to keep in mind the axiom, that it is not by the pulse, at the wrist and elsewhere, that we govern the body, but by the body that we govern the pulse. The work the heart has to perform is cut out for it by the capillaries, and to ease up the heart is easily done by easing up its burden to supply draught to the inward cremation.

In consequence of medical progress like that of the aforementioned articles of Solis-Cohen, Bishop and Morissey, the profession will have to grapple soon with the problem of the molecular action of the medicinal exhibit. From a scientific standpoint it is urgently indicated. But it can hardly be called rife. It is not a favorite topic. It threatens to jeopardize the practicability of all materia medica. If Hippocrates complained of lack of a safe bottom to stand on, the discoveries made since, although stupendous, do not seem to improve upon this particular. Chemistry has accommodated us with a wonderful insight into the nature of nutrition, and its distinction of nitrogenous substance and hydrocarbons seems has come to stay. But medicine, with particular reference to remedies, does not take much notice of the distinguishing classification. Our greatest writers dodge, as it were, the question. They shift the standpoint to suit the occasion. Bartholo, in speaking of coffee as a medicine, uses the style which, since Hippocrates, is classical, and speaking of it as a nutriment, he lets it parade as a chemical substance. Where to draw the line between medicine and nourishment the medical novice does not learn. All that he can go by is, that the one is bought at the druggist's the other at the grocer's. And to cap the climax, it is not much better with the distinction between unorganic and organic chemistry: If it was not for the bookbinder, who is so considerate as to bind the organic chemistry always at the end of the volume, at what of the chemical authorities could we learn which is which?

When a state of weak circulation is under discussion, so that the conclusion is arrived at to give a strengthening remedy, the exhibition of gluten may be decided on, and, as the reason, is given the tissue-forming qualities of the preparation. Supposing, however, a case of weak heart, so-called, the conclusion may be, to administer digitalis, etc., and, as the reason why, is stated the circumstance that digitalis and its allied group is a "stimulant." The celebrated J. R. Mayer, in his Mechanism of Warmth, speaks of the "magic influence" of alcohol upon "the nervous system and the mental functions." Where is the chemistry of that? A negro preacher in his sermons spoke of "intertranssubstantiationability," and when he was taken to account, he admitted that he did not know what he understood by the prolific vocabulation, but maintained that effect he had used that word to had been very "effervescent." Now, then, are the medical axioms to be sustained by similar polemics? If digitalis, alcohol, etc., do not benefit by being subservient to the reintegration of tissue in the regular metabolism, we must be furnished with the molecular specialty of their economisation in altogether exceptional affinity. The ordinary bonds of affinity which are based upon the equivalents of elementary proportion, would not hold, because they would do away with the entire specific medication, substitution of the same elements in *quod libet* other substance being professionally perfectly justified. If the simulation is not chemical, it must be mechanical, a sort of a "push," or "pull," or "thrust," and this it could not be demonstrated to be unless it was acknowledged a foreign substance, benefitting the diseased body by the disturbance it would create.

We do not wish to pass judgment on all the empiricism of materia medica. Chemistry itself, so far, is based on nothing but an hypothethis which may any day by new thought be overthrown. But if materia medica wants to hold its own, as a science, it should never lose sight of the truth, that, scientifically, the fact *that* a thing is, can never be maintained, unless it is demonstrated, *how* it is occurring. Mere professional elegance like the often used one, "In my hands the medicine did well," is hyperbolic, to say the least, especially in the aggravation which we once met with in a text-book of our friends, the homœopathic enemies, where the warning was made conspicuous by italics, "If you want to make sure to get the full benefit of this medicine, never exhibit it before half-past three in the afternoon."

